# High-sensitivity U-shaped biosensor for rabbit IgG detection based on PDA/AuNPs/PDA sandwich structure

**DOI:** 10.1515/nanoph-2025-0367

**Published:** 2025-12-01

**Authors:** Pengxiang Chang, Yirui Zhang, Ailing Zhang, Zhen Li, Zhiyang Wang, Yanmei Shi

**Affiliations:** Tianjin Key Laboratory of Thin Film Electronics and Communication Devices, Engineering Research Center of Optoelectronic Devices and Communication Technology, Ministry of Education, College of Integrated Circuit Science and Engineering, 66346 Tianjin University of Technology, Tianjin 300384, China

**Keywords:** surface plasmon resonance, U-shaped, gold nanoparticles, biosensor, rabbit IgG, polydopamine

## Abstract

Combining the advantages of polydopamine (PDA), gold nanoparticles (AuNPs), and U-shaped optical fibers, a PDA/AuNPs/PDA sandwich structure modified U-shaped optical fiber surface plasmon resonance (SPR) biosensor was proposed and used for the detection of rabbit IgG. The U-shaped structure significantly enhances the penetration depth of the evanescent field. The PDA/AuNPs/PDA sandwich structure boosts coupling efficiency of SPR and LSPR, increases the specific surface area of the sensor, and improves surface activity. The experimental results show that the refractive index (RI) sensitivity of the sensor is 6,206.32 nm/RIU in the RI range of 1.3353–1.3595, which is 1.63 times higher than the raw U-shaped sensor. The biosensor for detection of rabbit IgG achieved a biosensitivity of 0.153 nm/(ng/mL), which is two orders of magnitude higher and a limit of detection (LOD) of 0.131 ng/mL, which is one order of magnitude lower. The outstanding performance of the proposed biosensor offers a new solution for the detection of low-concentration biological solutions, and it has great potential applications in the field of medical diagnosis.

## 1 Introduction

Rapid and accurate detection of biomarkers is crucial for modern medical diagnostics, especially for early disease detection and personalized healthcare. Biosensors, which convert biological interactions into measurable signal, have become indispensable tools in the clinical and research fields due to their specificity, portability and real-time monitoring capabilities [1]. Among the traditional methods for biomolecular detection, electrochemical technique [2] is prone to passivation. Secondly, enzyme-linked immunosorbent assay (ELISA) [3] requires multiple incubation and washing steps, which are time-consuming. In contrast, label-free biosensors (such as surface plasmon resonance (SPR) and localized surface plasmon resonance (LSPR)) utilize the specific binding of antibodies to antigens to detect antigen molecules. This process is simple, controllable, and has high detection capabilities. Although SPR sensors have the above advantages, the LOD of sensor performance and detectable target types still needs to be improved.

The current methods for improving SPR sensor performance mainly include structural sensitization and material sensitization (coupling effects, surface activation and so on). The geometry of the optic fiber plays a key role to improve the sensor performance. Compared to conventional sensor configuration, the U-shaped structure significantly enhances the evanescent field intensity of the sensor surface [4]. Zhou and Yan (2022) developed MXene-based D-shaped SPR sensor, achieving refractive index (RI) sensitivity of 3,143 nm/RIU [5]. Although the D-shaped structure can improve the sensitivity of the sensor, its grinding process is uncontrollable and difficult to manufacture. Subsequently, Wei et al. introduced the S-taper structure into single-mode fiber, which increased the sensitivity to 3,244.62 nm/RIU [6]. However, this precision-dependent tapering technique has limitations in high-precision RI detection, and the sensor is difficult to manufacture. In contrast, Wang et al. introduced a U-shaped structure, which increased sensitivity to 3,808.74 nm/RIU [7]. Compared with the other two sensors, it is easy to manufacture and can achieve high-precision detection. Furthermore, it can be designed as a plug-and-play. However, while the U-shaped structure can significantly improve RI sensitivity of the sensor, the ability of this type of sensor to detect biomolecules is still limited by improvements in the functionalized membrane layer.

Recent advances in nanomaterial development have pioneered novel approaches for functionalized film modification in SPR sensors. LSPR generated by metallic nanoparticles has been widely used to enhance electric field confinement and improve RI sensitivity of optical fiber biosensor [8]. The coupling between SPR and LSPR can significantly enhance sensor performance [9]. For example, Wang et al. (2018) demonstrated a hybrid SPR/LSPR biosensor with AuNps, which significant increase RI sensitivity compared to normal SPR sensor [10]. Similarly, Ding et al. reported a novel sensitive SPR cortisol aptamer sensor based on the coupling effect of AuNPs and Au thin films, which showed a 40 % increase in sensitivity over the sensor without AuNPs modification [11]. From the above literature review, it can be seen that nanomaterial can significantly improve the physical properties of sensors, but they cannot enhance the surface activity of sensors. Therefore, it is necessary to introduce a type of biomaterial to enhance the surface activity of sensors.

To address these challenges, polydopamine (PDA) was applied to optic fiber SPR sensors. PDA self-polymerize into a functional film on sensor surfaces with abundant active sites [12], [13]. This film can immobilize AuNPs and adsorbing biomolecules, thereby enhance sensor RI sensitivity and surface reactivity [14]. Recent studies by Gao et al. have highlighted the dual role of PDA in improving sensor stability and acting as a substrate for antibody immobilization [15]. Cao et al. found that SPR sensors modified with PDA nanospheres achieved RI sensitivity of 2,427 nm/RIU, which is 55.7 % higher than raw sensor. When the sensor was used to detect C-reactive protein, a limit of detection (LOD) reached 0.22 μg/mL [16]. Zhang et al. (2025) developed an SPR sensor modified with Fe_3_O_4_@Au@PDA, which had an LOD of 0.61 ng/mL when detecting tebuconazole [17].

We proposed a PDA/AuNPs/PDA sandwich structure modified U-shaped optical fiber SPR biosensor. Compared with the raw sensor, the developed SPR biosensor exhibits higher sensitivity and low LOD. In addition, the PDA/AuNPs/PDA modified biosensor was used for the specific detection of rabbit IgG concentration. The evaluation and comparison of the fabricated biosensor with other reported SPR biosensors revealed the promising potential of our developed optical fiber SPR biosensor for the medical diagnosis.

## 2 Materials and methods

### 2.1 Materials

MMF (core/cladding layer diameter of 62.5/125 µm, purchased from Changfei Optical Fiber Company), AuNPs dispersion (particle size of 10 nm, concentration of 10 mg/mL), purchased from DEK Daojin, dopamine hydrochloride (DA, 98 %), bovine serum albumin (BSA) dry powder, rabbit IgG solution, goat anti-rabbit IgG protein solution, human IgG solution, phosphate buffer solution (PBS, 10 Mm), 10 mM Tris buffer (pH 8.9), which were purchased from Aladdin Co. 8.9.

### 2.2 Experimental methods

**Figure 1:**
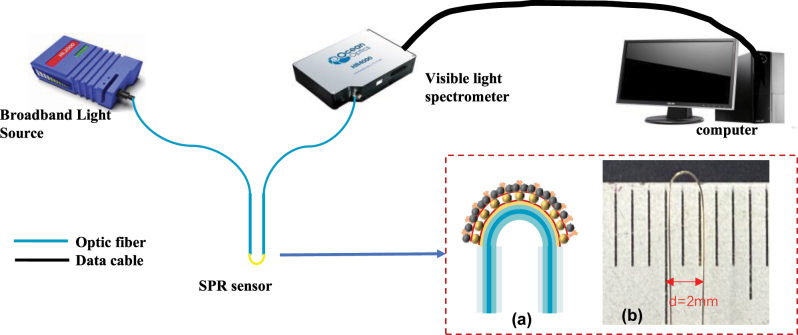
Experimental setup for optical fiber SPR biosensor: (a) Schematic diagram of U-shaped SPR sensor. (b) Physical diagram of U-shaped sensor with a bending diameter of 2 mm.

The optical fiber SPR biosensor is shown in Figure 1 The system consists of four components, a broadband light source (model HL2000, ocean optics, spectral range 200 nm–1,200 nm), an optic fiber SPR biosensor, a visible light spectrometer (model HR 4000, ocean optics, detection range 296 nm–1,080 nm with a resolution 0.02 nm) and a computer for signal demodulation, display and storage. Light from the broadband light source passes through the fiber SPR biosensor, exciting the SPR phenomenon and causing attenuation of the light signal at specific wavelengths. The attenuated light signal is detected by spectrometer and the SPR resonance wavelength is extracted from the minimum value of the normalized transmission spectrum using the centroid method.

### 2.3 Preparation of optical fiber SPR sensor

**Figure 2:**
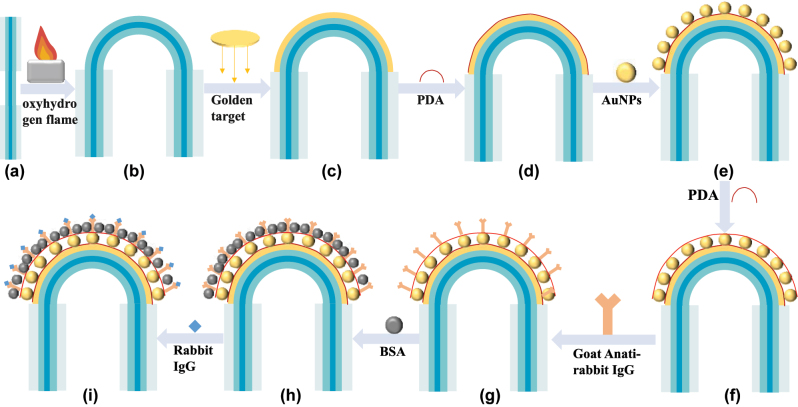
Schematic representation of the surface functionalization of the biosensor based on U-shape PDA/AuNPs/PDA structure: (a) MMF optical fiber after the removal of the coating layer. (b) The MMF is bent into a U-shape by heating it with a hydrogen-oxygen flame. (c) Sensor coated with an Au film. (d) Sensor modified with PDA. (e) Sensor modified with AuNPs. (f) Sensor modified with PDA. (g) Sensor coated with goat anti-rabbit IgG. (h) Blocking of the residual active sites on the SPR biosensor with BSA. (l) Fabrication of the biosensor for the specific detection of rabbit IgG.

The schematic diagram of preparation is shown in Figure 6 First, the coating layer of the MMF is removed to expose the cladding layer (as shown in Figure 2(a)). The exposed cladding layer is heated with hydrogen-oxygen flame to bend into a U-shaped structure with a diameter of 2 mm (as shown in Figure 1(b) and Figure 2(b)). The sensing area was coated with Au film by magnetron sputtering system. The thickness of Au film was 50 nm. (as shown in Figure 2(c)). To avoid the influence of impurities on the biological experiments, all instruments used in the experiments were thoroughly cleaned in an ultrasonic cleaner.

**Figure 3:**
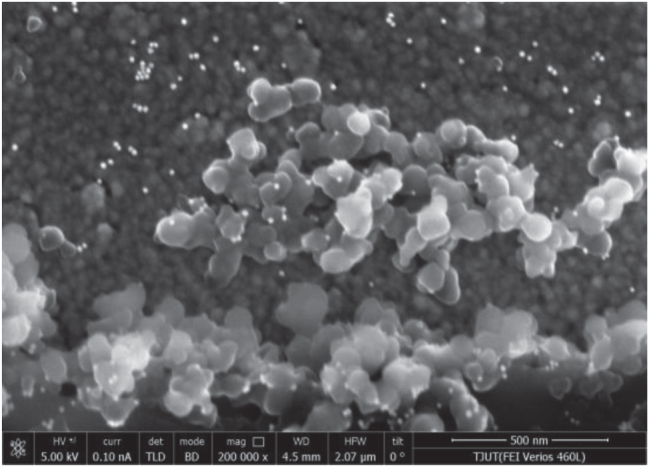
The scanning electron microscope image of the fabricated sensor.

**Figure 4:**
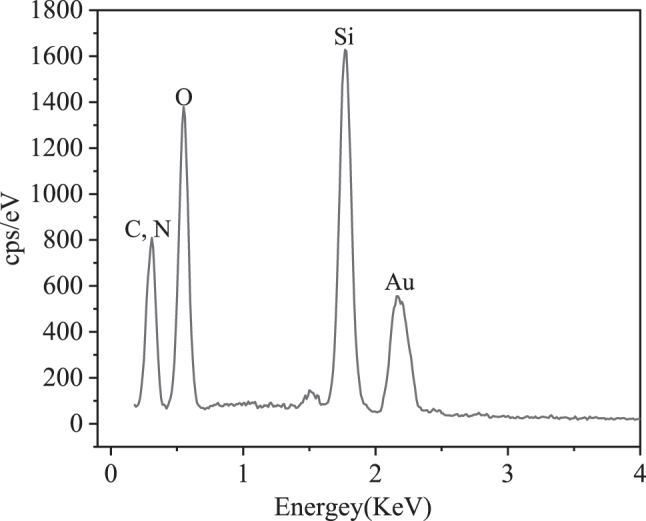
The energy dispersive X-ray spectrum (EDX) of the sensor surface.

To further modify AuNPs onto the sensor, the raw SPR sensor was first immersed in the PDA solution and placed in an incubator oscillating at 120 rpm. After 20 min of oscillation, the sensor was rinsed with deionized water and followed by air-drying to facilitate PDA on the sensor surface (as shown in Figure 2(d)). After that, the sensor was immersed in the AuNPs dispersion solution for 1 h and rinsed with deionized water (as shown in Figure 2(e)). Figure 3 is the scanning electron microscope image of the manufactured sensor. As can be seen from the figure, AuNPs are uniformly deposited on the sensor surface, with a particle size of approximately 10 nm. Figure 4 shows the energy dispersive X-ray spectrum of the sensor surface. The elements C and N were detected, indicating that PDA is fixed to the fiber surface.

The next step is fixing goat anti-rabbit IgG on the sensor surface. The sensor head was firstly coated by PDA film (as shown in Figure 2(f)) and then was immersed into goat anti-rabbit IgG solution (100 μg/mL of PBS buffer). After refrigeration at 4 °C for 24 h (as shown in Figure 2(g)), the sensor head was immersed in BSA solution (10 mg/mL) for 1 h to block remaining active sites on the sensor surface (as shown in Figure 2(h)). Finally, the sensor was immersed in different concentrations of rabbit IgG dilutions (diluted with PBS buffer) for 10 min at 37 °C (as shown in Figure 2(l)) to monitor the drift of the sensor resonance wavelength.

## 3 Results and discussion

### 3.1 Measurement of RI sensitivity

**Figure 5:**
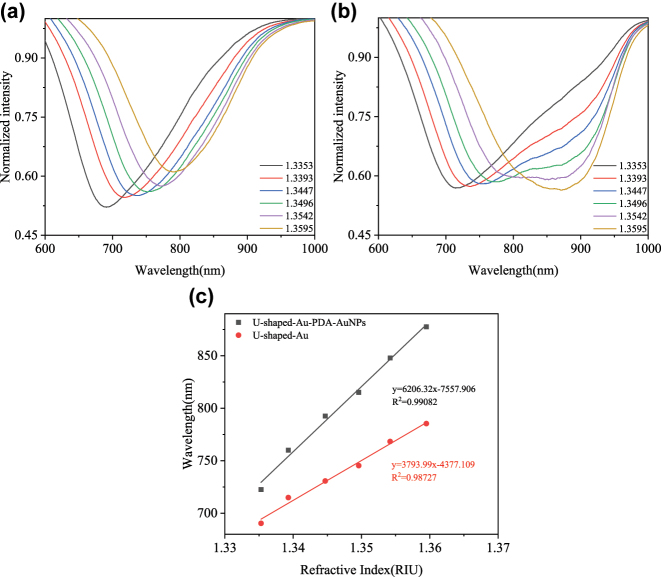
Performance characterization of the U-shaped SPR sensors: (a) Resonance spectra of U-shaped SPR sensor in different RI analytes, (b) Resonance spectra of U-shaped SPR sensor modified with AuNPs and PDA in different RI analytes, and (c) the resonance wavelength versus RI for both types of sensors.

In order to evaluate the performance of U-shaped sensor different RI value glycerol solutions (RI solutions of 1.3353, 1.3393, 1.3447, 1.3496, 1.3542, and 1.3595) were carried out at room temperature. The transmission spectra of the raw U-shaped sensor and the U-shaped sensor modified with PDA/AuNPs are shown in Figure 5(a) and (b). An increase in the refractive index leads to the phase-matching condition for the proposed multi-layer structure being satisfied at a longer wavelength. Figure 5(c) is the resonance wavelength versus RI for both types of sensors, the RI sensitivity of the sensor increased from 3,793.99 nm/RIU to 6,206.32 nm/RIU, which is 1.63 times higher than RI sensitivity of the raw U-shaped sensor. This sensitivity enhancement is caused by the enhanced electric field surrounding the sensor, which results from the coupling of SPR/LSPR and the high RI of PDA.

**Figure 6:**
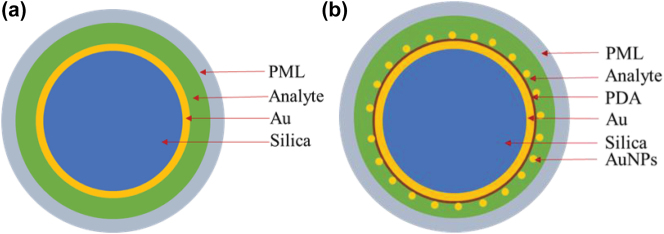
Schematic diagram of the sensor simulation structure: (a) Structural diagram of Au film-modified fiber SPR sensor, from outer to inner layers: 3.125 µm coreless optical fiber, 50 nm Au film and analyte. (b) Structural diagram of Au/PDA/AuNPs modified fiber SPR sensor, from outer to inner layers: 3.125 µm coreless optical fiber, 50 nm Au film, 20 nm PDA, AuNPs with a diameter of 20 nm and analyte.

**Figure 7:**
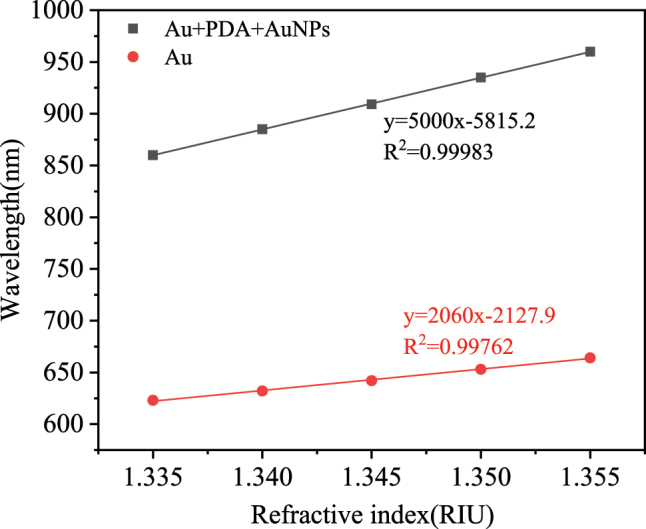
The resonance wavelength versus RI for both types of sensors.

The sensitivity enhancement by the PDA/AuNPs structure was also verified via simulations. The simulation structures without PDA/AuNPs and with PDA/AuNPs are schematically illustrated in Figure 6. The simulation results are shown in Figure 7. The results indicate that the RI sensitivity of the fiber optic SPR sensor with PDA/AuNPs is 2.43 times higher than that of the sensor without PDA/AuNPs. The results confirm that the sensitivity enhancement is caused by the enhanced electric field around the sensor, caused by the coupling of SPR/LSPR and the high RI of PDA.

### 3.2 Application for rabbit IgG specificity detection

**Figure 8:**
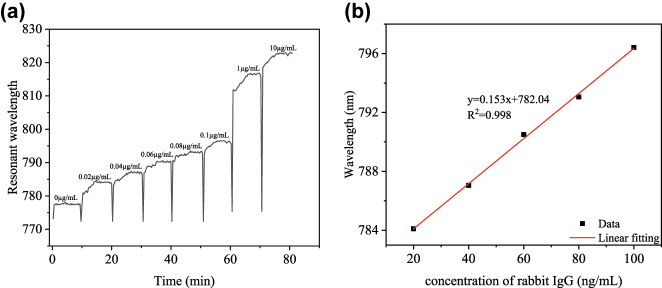
Detection performance for rabbit IgG using a U-shaped biosensor with a PDA/AuNPs/PDA sandwich structure: (a) SPR curves with time at different concentrations of rabbit IgG, (b) linear fit between rabbit IgG concentration and sensor resonance wavelength.

To evaluate the biosensitivity of the sensor, different concentrations of rabbit IgG solutions (0.02, 0.04, 0.06, 0.08, 1.00, 10.00 μg/mL) were injected into the reaction tube and the resonance wavelength was recorded in real time by a computer. When detecting for specific IgG concentration, the biosensor was rinsed three times with NaOH solution (10 mM) to wash away the antigen-antibody specific binding. As shown in Figure 8(a), the resonance wavelength of the sensor not only varied with the increase of rabbit IgG concentration, but also showed a gradual saturation response over time. This indicates that specific binding between rabbit IgG and goat anti-rabbit IgG is occurring and the immune response is gradually completed. The relationship between the concentration of rabbit IgG and the shift of the SPR curve is shown in Figure 8(b). The red line indicates the linear fit between the SPR curve and the rabbit IgG concentration (0.02–0.10 μg/mL). Within this linear fit range, the biosensor achieved a bio sensitivity of 0.153 nm/(ng/mL) for rabbit IgG with a fit coefficient of 0.998. The LOD was also used to evaluate the performance of the biosensor, which was defined as

(1) $$LOD=\frac{\lambda_{S}}{S_{bio}}$$

where *λ*_*S*_ represents the resolution of the spectrometer. The LOD of the biosensor was calculated as 0.131 ng/mL.

### 3.3 Specificity and repeatability studies

**Figure 9:**
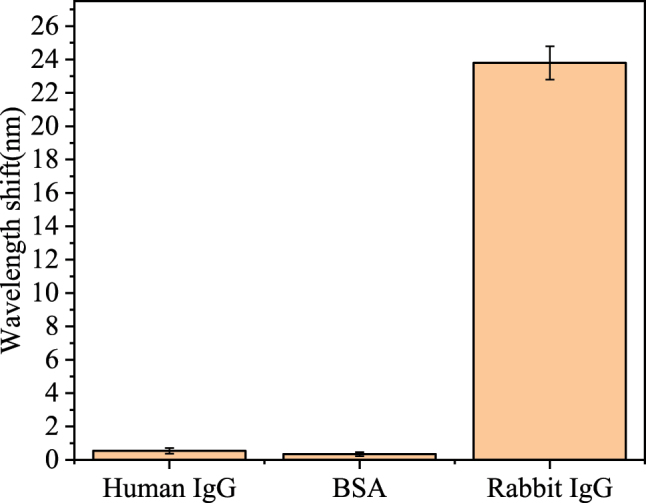
Resonance wavelength shifted IgG of human IgG, BSA and rabbit.

To assess the specificity of the PDA/AuNPs/PDA modified optic fiber SPR biosensor, BSA, human IgG and rabbit IgG solutions were used to evaluate the binding efficiency. Goat anti-rabbit IgG was used as a stationary antibody and protein solutions were introduced sequentially at a concentration of 100 μg/mL as a comparison experiment. Figure 9 illustrates that the resonance wavelength remained essentially unchanged when the interfering BSA and human IgG were detected. However, the wavelength shift corresponding to rabbit IgG was much higher than the other two, indicating that the sensor fulfils the purpose of specific detection. The excellent specificity of the biosensor based on PDA/AuNPs/PDA modification was demonstrated.

**Figure 10:**
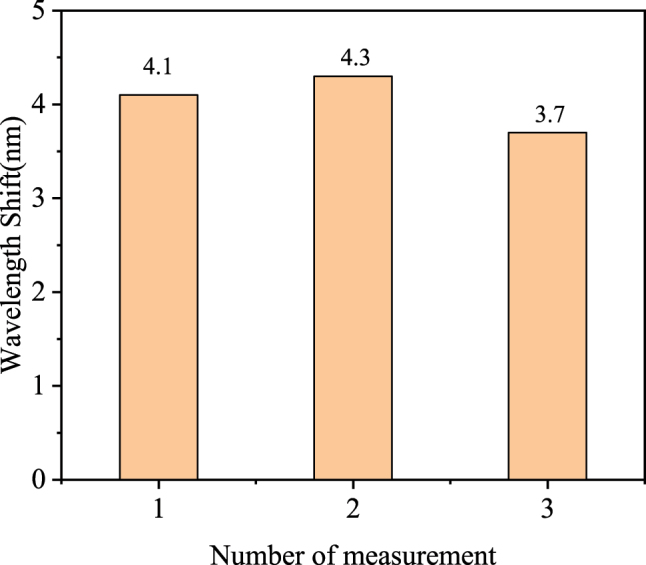
The repetitive performance experiments of the sensor.

The reusability of the sensor for 20 ng/ml rabbit IgG solution is shown in Figure 10 with NaOH solution cleaning between each measurement. It shows good repeatability. After 10 times NaOH solution cleaning, the Au film was observed to detach, which prevented further replication of the experiment. Reproducible detection may be in the future by improving the immobilization process of the sensor and the Au film.

### 3.4 Performance comparison of biosensors

The comparison between the proposed sensor and reported sensors in terms of biosensitivity and LOD is shown in Table 1. The biosensitivity of the proposed U-shaped biosensor is two orders of magnitude higher and the LOD is one order of magnitude lower than that of the biosensors in Table 1. This significant enhancement stems from the synergistic contributions of the U-shaped optical fiber architecture and the PDA/AuNPs/PDA sandwich structure. The U-shaped structure physically increases the penetration depth of the evanescent field. And the PDA/AuNPs/PDA sandwich structure not only improves the coupling efficiency between SPR and LSPR, also enhances surface reactivity through enlarging the functional surface area by PDA. In conclusion, the multilayered architecture on U-shaped MMF significantly enhances the RI sensitivity and biomolecular immobilization efficiency, which leads to an improved bio-sensitivity and a lower LOD for the proposed biosensor.

**Table 1:** Comparison of biosensor performance of different methods.

Methods used	Analyte	Structure	Material	Bio-sensitivity nm/(ng/mL)	LOD ng/mL	Ref.
LSPR	Rabbit IgG	SPR sensor	AuNR@SiO_2_	N.A.	12	[18]
	Goat-anti-human IgG	D-shaped PCF	AuNPs	N.A.	600	[19]
SPR	Human IgG	SPR sensor	Biotin-streptavidin sandwich integrated PDA-ZnO@Au	N.A.	37.5	[20]
	Human IgG	S-shaped SPR sensor	Au/TiO_2_	0.006025	1.66	[21]
	Human IgG	U-shaped MMF	MoS_2_/Au film/PDA	0.001014	19.7	[22]
	Rabbit IgG	POF	PNs	0.0014	15	[1]
	Goat-anti-rabbit IgG	POF	PDA-MoSe_2_@AuNPs-PDA	0.00037	54	[23]
	Human IgG	MSM	Ti_3_C_2_TxMXene/AuNPs/Au	0.0017	170	[24]
	Human IgG	POF	Ag@Au/GO	0.00053	37	[25]
	Rabbit IgG	U-shaped MMF	PDA/AuNPs/PDA	0.153	0.131	This work

## 4 Conclusions

A highly sensitive U-shaped SPR biosensor is demonstrated in this paper, which utilizes the synergistic enhancement of the U-shaped optical fiber architecture and the PDA/AuNPs/PDA sandwich structure. The biosensor for detection of rabbit IgG achieved a biosensitivity of 0.153 nm/(ng/mL), which is two orders of magnitude higher and LOD of 0.131 ng/mL, which is one order of magnitude lower. The significantly improved performance of the proposed biosensor enables potential applications in medical diagnosis.
